# Research focus and trends of the association between gut microbiota and neuroinflammation

**DOI:** 10.3389/fmicb.2025.1564717

**Published:** 2025-06-03

**Authors:** Shan Wu, Nanjie Chen, Chuanchi Wang, Kwok-Fai So

**Affiliations:** ^1^Guangdong-Hong Kong-Macau Institute of CNS Regeneration, Jinan University, Guangzhou, China; ^2^Xin-Huangpu Joint Innovation Institute of Chinese Medicine, Guangzhou, China; ^3^Institute of Grand Scientific Devices, Beijing University of Aeronautics and Astronautics, Beijing, China; ^4^Modern Traditional Chinese Medicine Haihe Laboratory, Tianjin University of Traditional Chinese Medicine, Tianjin, China

**Keywords:** gut microbiota, neuroinflammation, bibliometric analysis, research trends, neurodegenerative

## Abstract

**Background:**

The interaction between the gut microbiota and neuroinflammation plays a crucial role in the pathogenesis of many diseases, particularly neurodegenerative diseases, and has become one of the focal points of research in recent years. Despite the large number of related studies, there is currently a lack of comprehensive analysis and prediction of these data to drive the field forward. This study aims to systematically analyze the clinical practices and research hotspots of the underlying mechanisms in this field using bibliometric and visualization methods, and to explore the future development pathways.

**Methods:**

CiteSpace, VOSviewer, GraphPad Prism and other software were used to analyze 1,404 studies on gut microbiota and neuroinflammation collected by the core of the Web of Science since 2000, to visually present the collaborative network between literatures, structure of authors and countries, co-occurrence of keywords, emerging reference literature, and research hotspots.

**Results:**

From 2000 to 2024, the number of related papers on this topic showed an overall upward trend, and the annual citation peaked in 2020, with significant contributions from China and the United States. Research focused on the relationship between gut microbiota and neuroinflammation, with a particular emphasis on investigating the mechanisms of the microbiota-gut-brain axis through both basic and clinical research. Treatment strategies include probiotic therapy, fecal microbiota transplantation and traditional Chinese medicine.

**Conclusion:**

This study comprehensively reviews the research progress on the association between gut microbiota and neuroinflammation, and discusses the current research focus and frontier directions of this relationship, so as to provide reference for the development of this field.

## 1 Introduction

The interaction between gut microbiota and their hosts has emerged as a prominent research area in recent years. Mounting evidence suggests that maintaining a balanced composition of gut microbial communities is crucial for the overall health of the host organism. In addition to its role in food digestion and absorption, the gut microbiota also exerts influence on brain function and behavior through the intricate network known as the microbiota-gut-brain axis (MGBA). The gut microbiota communicates with the brain through the “gut-brain axis, “a sophisticated network that not only encompasses the production of microbial metabolites such as short-chain fatty acids (SCFAs) and neurotransmitters, but also orchestrates immune system function to modulate neuroinflammation (Chidambaram et al., 2022).

Recently, the bidirectional effects of the gut microbiota on central nervous system (CNS) inflammation through the MGBA mechanism have been gradually elucidated. Previous studies have demonstrated a close association between dysbiosis of the gut microbiota and the development and occurrence of various neuroinflammatory-related diseases. Specifically, patients with Alzheimer’s disease (AD), Parkinson’s disease (PD), and multiple sclerosis (MS) often exhibit significantly distinct composition and functionality of their gut microbiota compared to healthy individuals (Zhang et al., 2024; Nuzum et al., 2024). The study revealed that dysbiosis of the gut microbiota can influence neuroinflammation through multiple mechanisms, such as but not limited to the disruption of intestinal barrier function, the alteration of metabolic products, and the abnormal activation of the immune system (Kumari et al., 2023). The interactions of these mechanisms might further intensify neuroinflammation, thus facilitating the pathological advancement of neurodegenerative diseases. Additionally, the metabolic products of the gut microbiome, such as SCFAs, have been demonstrated to play a vital role in regulating the host immune response and maintaining the integrity of the gut barrier as well as the blood–brain barrier (BBB) (Ashique et al., 2024; Mathias et al., 2024). SCFAs can regulate the expression of inflammatory factors by binding to receptors on host cells, thereby influencing the development of neuroinflammation. Hence, the equilibrium of the gut microbiome is potentially of great significance in preventing and treating neurodegenerative diseases.

Bibliometric analysis is a quantitative approach for studying scientific literature, which can objectively disclose the potential connections and inherent laws among research objects by excavating a large amount of literature data. Therefore, this study undertakes a bibliometric analysis on the literature related to the gut microbiome and neuroinflammation published since 2000, to reveal the knowledge structure of the field for the first time, identify key research themes and hot topics, disclose the latest advancements and significant findings in these research areas, and analyze the directions where future research might develop. Through this study, we anticipate providing a new perspective on understanding the complex relationship between the gut microbiome and neuroinflammation, and offer a reference for future research endeavors.

## 2 Methods

### 2.1 Data sources and cleaning

**Figure 1:**
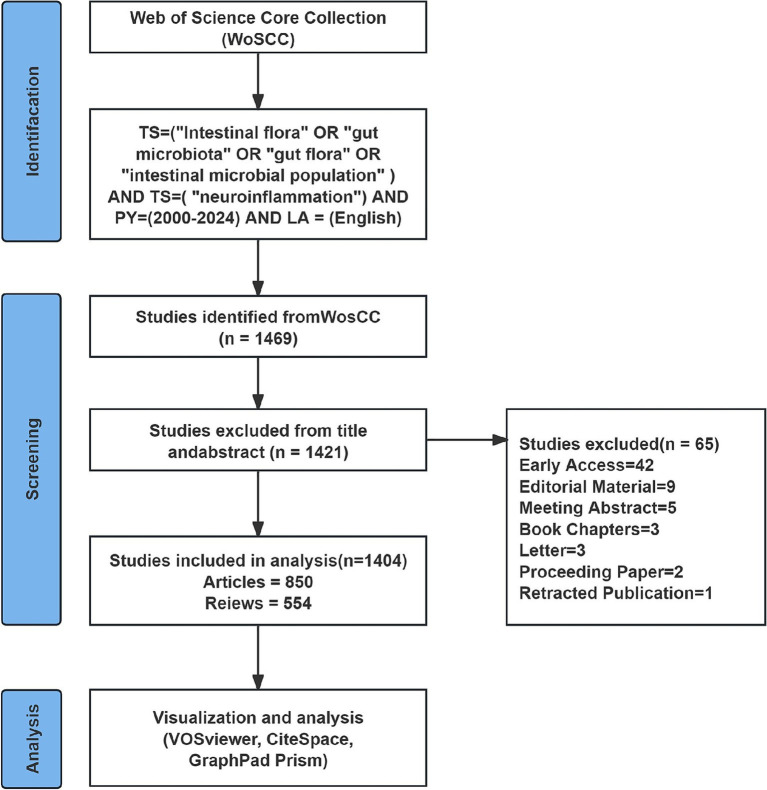
Publication’s screening flowchart.

This study used CiteSpace, VOSviewer, and GraphPad Prism software to carry out visual analysis of the selected literature. An exploration of the Web of Science Core Collection (WoSCC) literature database was conducted on September 5, 2024. The search formula was “TS = (“Intestinal flora” OR “gut microbiota” OR “gut flora” OR “intestinal microbial population”) AND TS = (“neuroinflammation”) AND PY = (2000–2024) AND LA = (English),” with the time interval ranging from 2000-01-01 to 2024-09-05. The exported document records consist of titles, authors, institution names, keywords, abstract, journal, publication time, etc. To more precisely target the information requisite for the topic, this study solely incorporates literature of the document types “articles” and “review.” Import the documents into EndNote for preliminary deduplication. Then, two authors (S W and NJ C) manually compare the titles, abstracts, keywords and other contents to eliminate the following literatures: (1) Literatures with duplicated titles and abstracts; (2) Literature that is inconsistent with the target research content; and (3) Literature of types such as conference proceedings, edited materials, letters, conference abstracts, book reviews, corrections, data papers, etc. After data cleaning, all the literature information was exported in plain text format. The process of including literature is presented in Figure 1.

### 2.2 Data analysis and visualization

This study conducted a visual analysis of the selected literature using CiteSpace, VOSviewer, and GraphPad Prism software. In CiteSpace, the following parameter settings were adopted: (1) The time range was from January 1, 2000 to September 5, 2024, with the analysis time unit set as 1 year; (2) Threshold filtering was applied to select the top *n* authors with the highest frequency in each time period (*n* = 50) to highlight important literature; (3) Node types selected for visualization analysis included country, institution, author, and keyword, generating co-occurrence graphs; and (4) Pruning, trimming the functional area to highlight the connection graph of important nodes; other parameters were set to the default values of the system. In VOSviewer, we selected the minimum document quantity of nodes according to the needs of data visualization, and set other documents to the default values. We analyzed the distribution of annual publications and used GraphPad Prism to draw publication data tables and tables of publications by country/region. CiteSpace and VOSviewer generated author, country/region, journal, keyword, and reference collaboration networks, co-occurrence networks, and co-citation graphs of references.

## 3 Results

### 3.1 Global trend in publication outputs and citations

**Figure 2:**
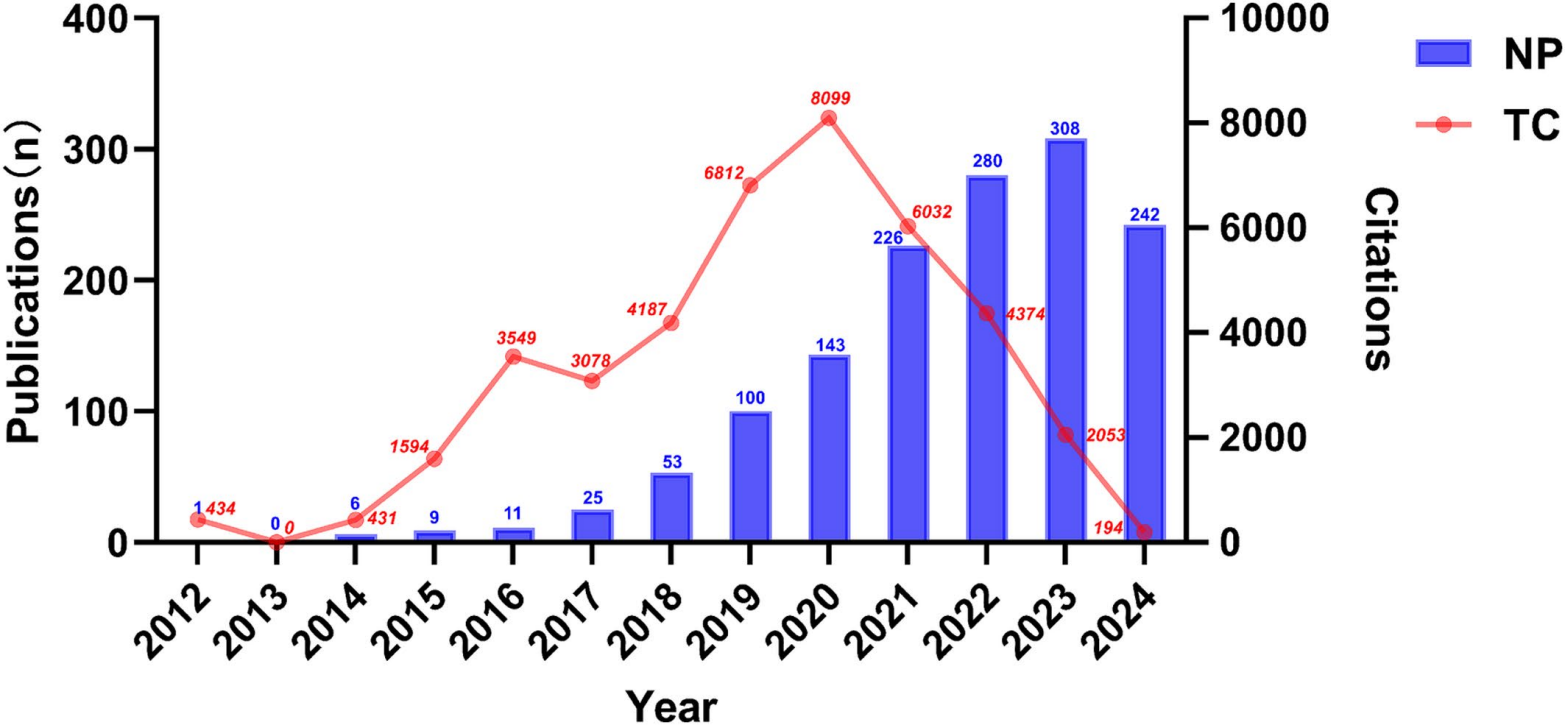
Temporal trends in publications of the association between gut microbiota and neuroinflammation, as indicated by the number of publications (NP) and total citation (TC).

A total of 1,404 articles examining the correlation between gut microbiota and neuroinflammation were included, comprising 850 original studies and 554 reviews. Figure 2 illustrates the yearly publication volume and citation frequency of relevant publications from January 2000 to September 2024. Overall, there has been a consistent upward trend in annual publications on gut microbiota and neuroinflammation, with a notable surge since 2019. Annual citations reached their peak in 2020, while the number of publications continued to grow, reaching its zenith in 2023. It is anticipated that by the end of 2024, the number of publications in this field will surpass previous years.

### 3.2 Country and institution analysis

**Figure 3:**
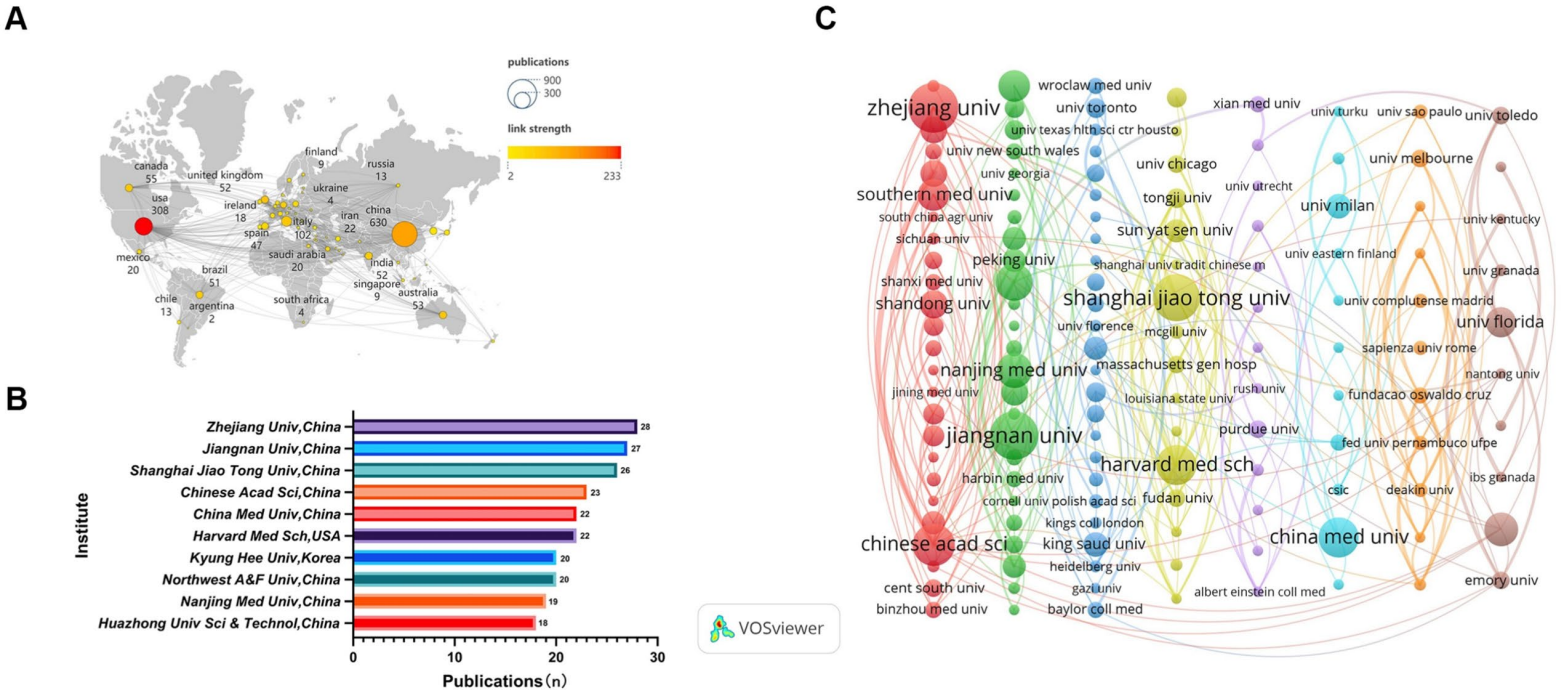
Analysis of country/region. **(A)** Visual clustering analysis of the national collaboration network. **(B)** The top 10 institutions by publication volume. **(C)** Visual clustering analysis of inter-institutional collaboration relations. Nodes represent countries/institutions, the size reflects the number of publications, the connecting lines indicate cooperation, the thickness represents the degree of closeness, and different colors represent different clusters, which can reflect the size and composition of the clusters.

A total of 78 countries and 1,000 academic institutions were involved in the research on the association between gut microbiota and neuroinflammation. China (*n* = 630) made the greatest contribution to the number of publications, followed by the United States (*n* = 308) and Italy (*n* = 102). By filtering and visualizing the 78 countries based on the number of publications equal to or greater than 2, and constructing a collaboration network based on the number of published papers and relationships in each country. There was close collaboration among different countries (Figure 3A). For instance, the United States collaborated closely with China, Brazil, Italy, and Canada, while China collaborated closely with the United States and Australia.

Among the top 10 academic institutions worldwide in terms of the number of publications regarding the relationship between gut microbiota and neuroinflammation, three countries are represented. Eight of them are from China, while the remaining two are from the United States and South Korea. The top three academic institutions with the greatest number of publications on this subject are Zhejiang University (*n* = 28), Jiangnan University (*n* = 27), and Shanghai JiaoTong University (*n* = 26) (Figure 3B). Subsequently, 151 academic institutions were selected based on the criterion that the minimum number of publications was equal to 5, and they were visualized based on the number of publications and relationships. The collaboration among Chinese Academy of Sciences, University of Chinese Academy of Sciences and ‌South China Agricultural University was highly intense, and the same gone for Harvard University, Tongji University and Sun Yat-Sen University (Figure 3C).

### 3.3 Journals and co-cited journals

Research on the association between gut microbes and neuroinflammation has been published in 443 journals. The most published papers are Nutrients (*n* = 52, 11.74%), followed by International Journal of Molecular Sciences (*n* = 42, 9.48%), Brain Behavior and Immunity (*n* = 38, 8.58%). Among them, the highest impact factor was found in the Journal of Neuroinflammation (IF: 9.3). This was followed by Brain Behavior and Immunity (IF: 8.8) and Biomedicine & Pharmacotherapy (IF: 6.9). Of the top 10 journals cited, five were cited more than 2,000 times, with Scientific Reports (2,606 times) being the most cited journal, followed by Plos One (2,600 times) (Table 1).

**Table 1:** The top 10 most productive journals and co-cited journals.

Rank	Journal	Counts	IF (2024)	JCR (2024)	Co-cited journal	Co-citation	IF (2024)	JCR (2024)
1	Nutrients	52	4.8	Q1	Scientific Reports	2,606	3.8	Q2
2	International Journal of Molecular Sciences	42	4.9	Q2	Public Library of Science ONE	2,600	3.8	Q2
3	Brain Behavior and Immunity	38	8.8	Q1	Nature	2,413	50.5	Q1
4	Frontiers in Immunology	36	5.7	Q1	Brain, Behavior, and Immunity	2,260	8.8	Q1
5	Frontiers in Neuroscience	30	3.2	Q2	Proceedings of the National Academy of Sciences of the United States of America	2,151	9.4	Q1
6	Journal of Agricultural and Food Chemistry	26	5.7	Q1	Cell	1,972	45.5	Q1
7	Food & Function	24	5.1	Q1	Nutrients	1,873	4.8	Q1
8	Journal of Neuroinflammation	24	9.3	Q1	International Journal of Molecular Sciences	1,818	4.9	Q2
9	Frontiers in Pharmacology	21	4.4	Q2	Journal of Neuroinflammation	1,801	9.3	Q1
10	Biomedicine & Pharmacotherapy	20	6.9	Q1	Journal of Alzheimer’s Disease	1,732	3.4	Q2

**Figure 4:**
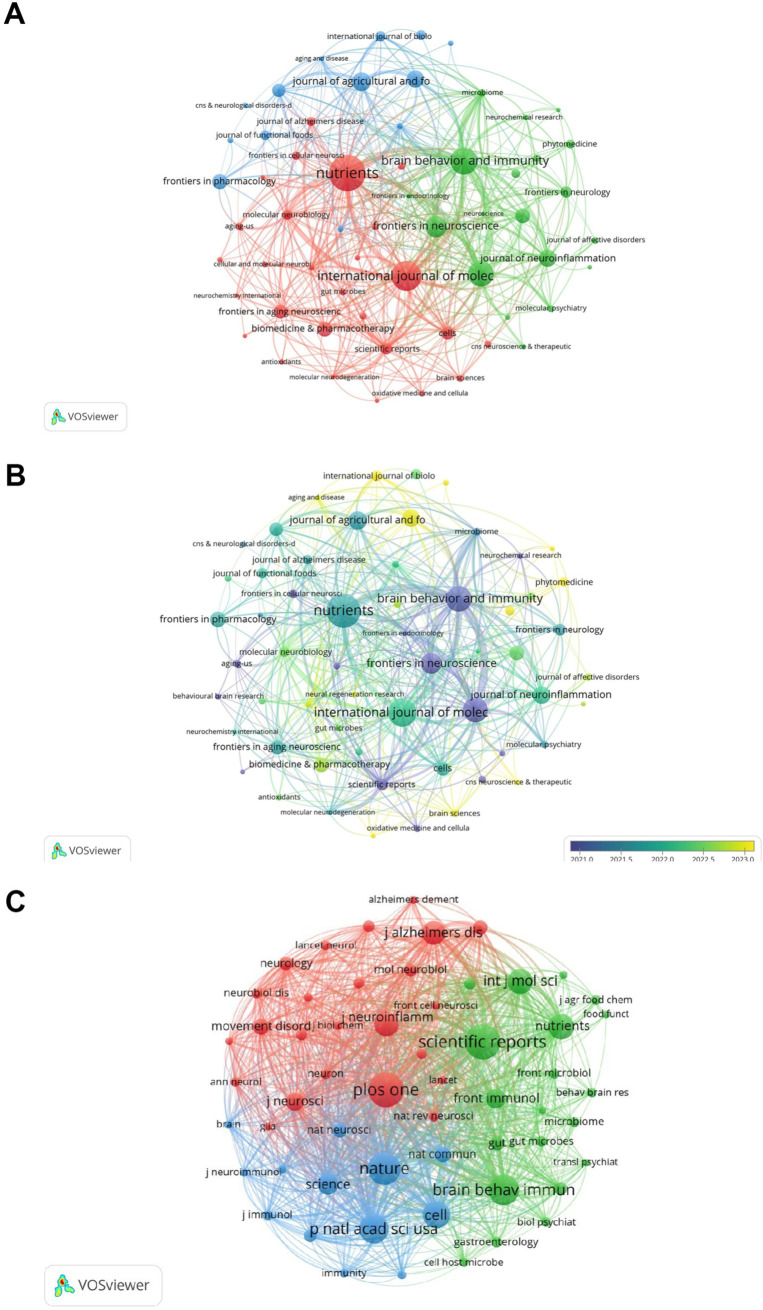
Analysis of journals. **(A)** Visualization of journal collaboration network. **(B)** Visualization of journal collaboration network with overlaid time. **(C)** Co-citation network visualization. The different colors in the collaborative network diagram represent different clusters, which can reflect the size and composition of the clusters. In the superimposed temporal collaborative network diagram, the time axis from purple to yellow indicates the emergence time and development sequence of these hotspots. The same applies to Figures 5–7.

Subsequently, we selected 59 journals based on the principle of a minimum co-cited publication number of 6 and created a journal network diagram. Figures 4A,B reveal that the citation relationships between Nutrients and International Journal of Molecular Sciences, as well as Brain Behavior and Immunity, are vigorous. Among the co-cited journals, the highest impact factor belongs to Nature (IF: 50.5), followed by Cell (IF: 45.5). Then, we chose the journals with a maximum co-cited number of more than 500 times and drew a co-cited network diagram. Figure 4C indicates that the co-cited relationships between Plos one and Nature, Scientific Reports, Cell, and Proceedings of the National Academy of Sciences of the United States of America are dynamic.

### 3.4 Author productivity and co-citation analysis

**Figure 5:**
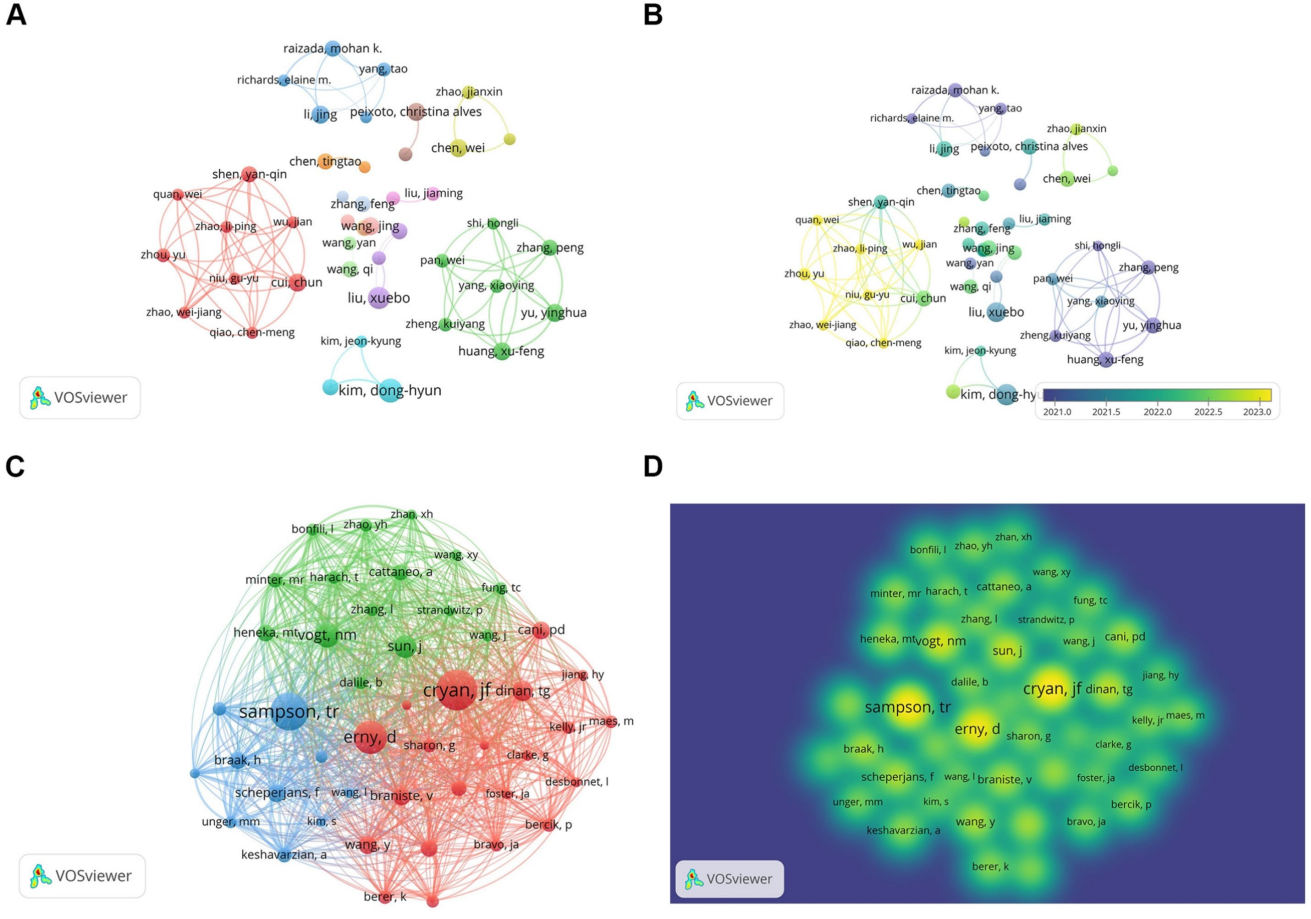
Author and co-cited author analysis. **(A)** Author collaboration network visualization. **(B)** Author collaboration network visualization with overlaid time. Colors represent average time. **(C)** Co-cited author network diagram. **(D)** Co-cited author density map.

A total of 8,194 authors engaged in research on the relationship between gut microbiota and neuroinflammation. Among the top 10 authors, two of them each published over 10 papers (Table 2). We established a cooperation network based on the criterion that authors had published at least 6 papers in this field (Figures 5A,B). Three small cooperation networks were formed around Cui Chun, Li Jing, and Yu Yinghua, with relatively fewer researchers collaborating with others, indicating that the current cooperation network structure in this area is relatively scattered and has not yet formed a large-scale cooperation network.

**Table 2:** The top 10 most productive authors.

Rank	Authors	Institute, Country	NP	TC
1	Kim, Dong-Hyun	Kyung Hee University, Korea	12	283
2	Liu, Xuebo	Northwest A&F University, China	11	436
3	Chen, Wei	Naval Medical University, China	9	469
4	Cui, Chun	Jiangnan University, China	9	102
5	Huang, Xu-Feng	Xuzhou Medical University, China	9	134
6	Li, Jing	Chinese Academy of Sciences, China	9	212
7	Peixoto, Christina Alves	Aggeu Magalhães Institute, Brazil	9	513
8	Wang, Jing	Henan University of Chinese Medicine, China	9	495
9	Yu, Yinghua	Xuzhou Medical University, China	9	122
10	Zhang, Xin	Lanzhou University, China	12	144

NP, number of publications; TC, total citations.

Among the 58,786 co-cited authors, those with a minimum co-citation count of 100 or above were selected, and a co-cited network was plotted (Figures 5C,D). There were three authors whose co-citation counts exceeded 300, namely Cryan, JF (*n* = 424), Sampson, TR (*n* = 394), and Erny, D (*n* = 345), and they exhibited close co-cited relationships.

### 3.5 Co-cited references analysis

**Figure 6:**
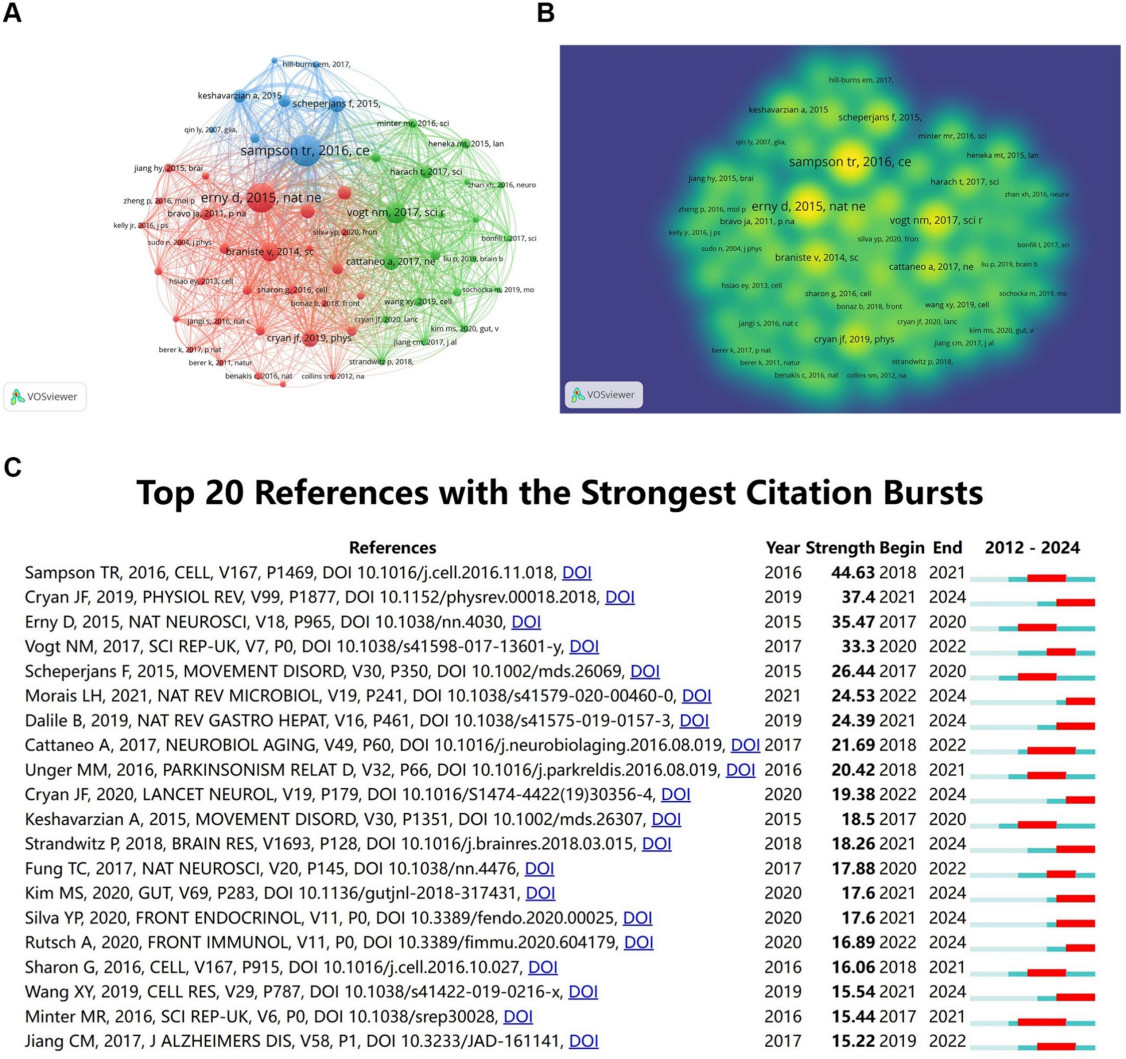
Co-cited references analysis. **(A)** Co-cited references network. **(B)** Co-cited references density map. **(C)** Top 20 references with the strongest citation bursts.

Over the past 20 years, a total of 87,577 citations have been made to research on the role of gut microbiota in neuroinflammation. Among the top 10 most highly cited papers, at least 128 citations have been received, with three papers being cited over 200 times. We constructed a co-citation network graph using papers with a co-citation count of at least 60. Sampson (2016) in Cell, Vol. 167, P1469, Erny et al. (2015) in Nat Neurosci, Vol. 18, P965, Braniste (2014) in Sci Transl Med, Vol. 6, and Vogt (2017) in Sci Rep-UK, Vol. 7, have a considerable number of co-citations, indicating that these three papers are frequently cited together (Figures 6A,B).

The citation co-occurrence map (Figure 6C) generated by CiteSpace reveals that the reference with the most intense citation burst (intensity = 44.63) is “Gut Microbiota Regulate Motor Deficits and Neuroinflammation in a Model of Parkinson’s Disease” by Sampson et al. (2016). The period of citation burst is from 2018 to 2021. The second most intense citation burst (intensity = 37.4) is “The Microbiota-Gut-Brain Axis” by Cryan JF et al., whose citation burst period ranges from 2021 to 2024 in Physiological Reviews. We have summarized the main research contents of the top 20 most-cited papers with the strongest citation bursts in Table 3.

**Table 3:** The top 20 references with the strongest citation bursts.

Rank	Title	Key points	Journal	Strength	Year
1	Gut Microbiota Regulate Motor Deficits and Neuroinflammation in a Model of Parkinson’s Disease	The study explored how the gut microbiome regulates motor deficits and neuroinflammation in PD models, revealing a functional connection between the gut microbiome and PD.	Cell	44.63	2016
2	The Microbiota-Gut-Brain Axis	A comprehensive review of the research progress of MGBA was carried out, exploring how the gut microbiome can influence brain function and behavior through diverse pathways, thereby affecting the host’s physiological, behavioral, and cognitive functions and playing a significant role in various neuropsychiatric disorders.	Physiological Reviews	37.4	2019
3	Host microbiota constantly control maturation and function of microglia in the CNS	The study probed into the influence of the host microbiome on CNS microglia and discovered that the microbiome governs the maturation and function of microglia via metabolites such as SCFAs. The absence of the microbiome gives rise to abnormal microglia, while reintroducing a complex microbiome can partially restore its normal traits.	Nature Neuroscience	35.47	2015
4	Gut microbiome alterations in Alzheimer’s disease	The composition of gut microbiota in AD patients was investigated, and it was discovered that a significant difference existed between AD patients and healthy control groups, indicating that gut microbiota might play a role in the pathogenesis of AD.	Scientific Reports	33.3	2017
5	Gut microbiota are related to Parkinson’s disease and clinical phenotype	By comparing the fecal microbiome of PD patients to that of healthy controls, it was discovered that the composition of the intestinal microbiome and its correlation with clinical phenotypes in PD patients were notably different from those in healthy controls.	Movement Disorders	26.44	2015
6	The gut microbiota–brain axis in behavior and brain disorders	This paper reviewed the new evidence regarding the complex and crucial links between the gut microbiome and the brain, which involve multiple biological systems, as well as the potential contribution of the gut microbiome to neurological disorders.	Nature Reviews Microbiology	24.53	2021
7	The role of short-chain fatty acids in microbiota–gut–brain communication	The role of SCFAs in the communication between the microbiome-gut-brain was investigated, and how SCFAs mediate the interaction between the microbiota-gut-brain to influence psychological functions, emotions, and cognitive processes was explored.	Nature Reviews Gastroenterology & Hepatology	24.39	2019
8	Association of brain amyloidosis with pro-inflammatory gut bacterial taxa and peripheral inflammation markers in cognitively impaired elderly	The study investigated the association of brain amyloid deposition with pro-inflammatory intestinal bacterial microbiota and peripheral inflammatory markers in elderly individuals suffering from cognitive impairment.	Neurobiology of Aging	21.69	2017
9	Short chain fatty acids and gut microbiota differ between patients with Parkinson’s disease and age-matched controls	The study compared the gut microbiome and the levels of short-chain fatty acids (SCFA) between patients with Parkinson’s disease (PD) and age-matched controls, and discovered that PD patients had distinct concentrations of SCFA in their stool and variations in the abundance of certain bacterial phyla and genera.	Parkinsonism & Related Disorders	20.42	2016
10	The gut microbiome in neurological disorders	The potential role of the gut microbiome in diverse neurological disorders has been investigated, and the likelihood of the microbiome being a susceptibility factor for these diseases has been examined, along with the direction of future research and therapeutic strategies.	The Lancet Neurology	19.38	2020
11	Colonic bacterial composition in Parkinson’s disease	The composition of the microbiome in the sigmoid colon of PD patients was investigated, and it was discovered that there was a significant disparity between the PD group and the healthy control group. It was hypothesized that these alterations might trigger inflammation, resulting in the misfolding of alpha-synuclein and thereby facilitating the pathological progression of PD.	Movement Disorders	18.5	2015
12	Neurotransmitter modulation by the gut microbiota	This review presented a summary of the role of the gut microbiome in regulating neurotransmitters, along with its potential influence on MGBA communication and therapeutic uses.	Brain Research	18.26	2018
13	Interactions between the microbiota, immune and nervous systems in health and disease	This review elaborated on the roles of CNS residency and peripheral immune pathways in MGBA in health and neurological disorders.	Nature Neuroscience	17.88	2017
14	Transfer of a healthy microbiota reduces amyloid and tau pathology in an Alzheimer’s disease animal model	The study explored the effects of FMT from healthy mice on the pathological alterations of amyloid beta and tau proteins in the brains of AD mice, as well as the causal connection between the gut microbiome and the pathogenesis of AD.	Gut	17.6	2020
15	The Role of Short-Chain Fatty Acids from Gut Microbiota in Gut-Brain Communication	This review presented a summary of the research on the interaction between the intestinal microbiome and the brain mediated by SCFAs, and investigates their role in neuro-immune-endocrine regulation as well as their potential influence on the treatment of CNS diseases.	Frontiers in Endocrinology	17.6	2020
16	The Gut-Brain Axis: How Microbiota and Host Inflammasome Influence Brain Physiology and Pathology	The discussion centered on how the gut microbiome and its metabolites could impact brain physiology and pathology via the MGBA and inflammasome pathways, particularly in the cases of MS, AD, PD, and neuropsychiatric disorders.	Frontiers in Immunology	16.89	2020
17	The Central Nervous System and the Gut Microbiome	The discussion focused on how the gut microbiome affects neural development and function via molecular signals, investigating its vital role in the growth of the nervous system and the equilibrium of mental health and disease.	Cell	16.06	2016
18	Sodium oligomannate therapeutically remodels gut microbiota and suppresses gut bacterial amino acids-shaped neuroinflammation to inhibit Alzheimer’s disease progression	The study probed into how gut dysbiosis facilitates the accumulation of phenylalanine and isoleucine, resulting in neuroinflammation, which expedites the advancement of AD. It was found that sodium oligomannoside can reshape the gut microbiome to inhibit this inflammatory response and enhance cognitive function.	Cell Research	15.54	2019
19	Antibiotic-induced perturbations in gut microbial diversity influences neuro-inflammation and amyloidosis in a murine model of Alzheimer’s disease	The study probed into how changes in gut microbial diversity induced by long-term antibiotic treatment affect the Aβ amyloid pathology and neuroinflammation in AD mouse models.	Scientific Reports	15.44	2016
20	The Gut Microbiota and Alzheimer’s Disease	This review summarized the potential role of the gut microbiome in the advancement of AD and investigated how gut microbiome dysbiosis may accelerate the pathological progression of AD through mechanisms such as influencing MGBA, enhancing intestinal and BBB permeability, and triggering inflammation.	Journal of Alzheimer’s Disease	15.22	2017

### 3.6 Keywords analysis of research hotspots

#### 3.6.1 Co-occurrence analysis of keywords

By conducting co-occurrence analysis on keywords, it becomes feasible to promptly identify the research hotspots in a specific field. In the case of the research regarding the role of gut microbiota in neuroinflammation, there are a total of 4,867 keywords. Table 4 presents the top 20 keywords with the highest frequency. Besides the theme words, Alzheimer’s disease, inflammation, and Parkinson’s disease occur most frequently, representing the main research directions in studies related to gut microbiota and neuroinflammation.

**Table 4:** The top 20 co-occurrence key words.

Rank	Keywords	Counts	Rank	Keywords	Counts
1	Neuroinflammation	783	11	Oxidative stress	183
2	Gut microbiota	767	12	Chain fatty-acids	144
3	Alzheimer’s disease	355	13	Probiotic	127
4	Inflammation	346	14	Activation	125
5	Parkinson’s disease	244	15	Dysbiosis	116
6	Brain	224	16	Model	111
7	Microglia	211	17	Depression	107
8	Gut-brain axis	202	18	Expression	103
9	Microbiota	200	19	Intestinal microbiota	100
10	Mouse model	192	20	Neurodegeneration	97

**Figure 7:**
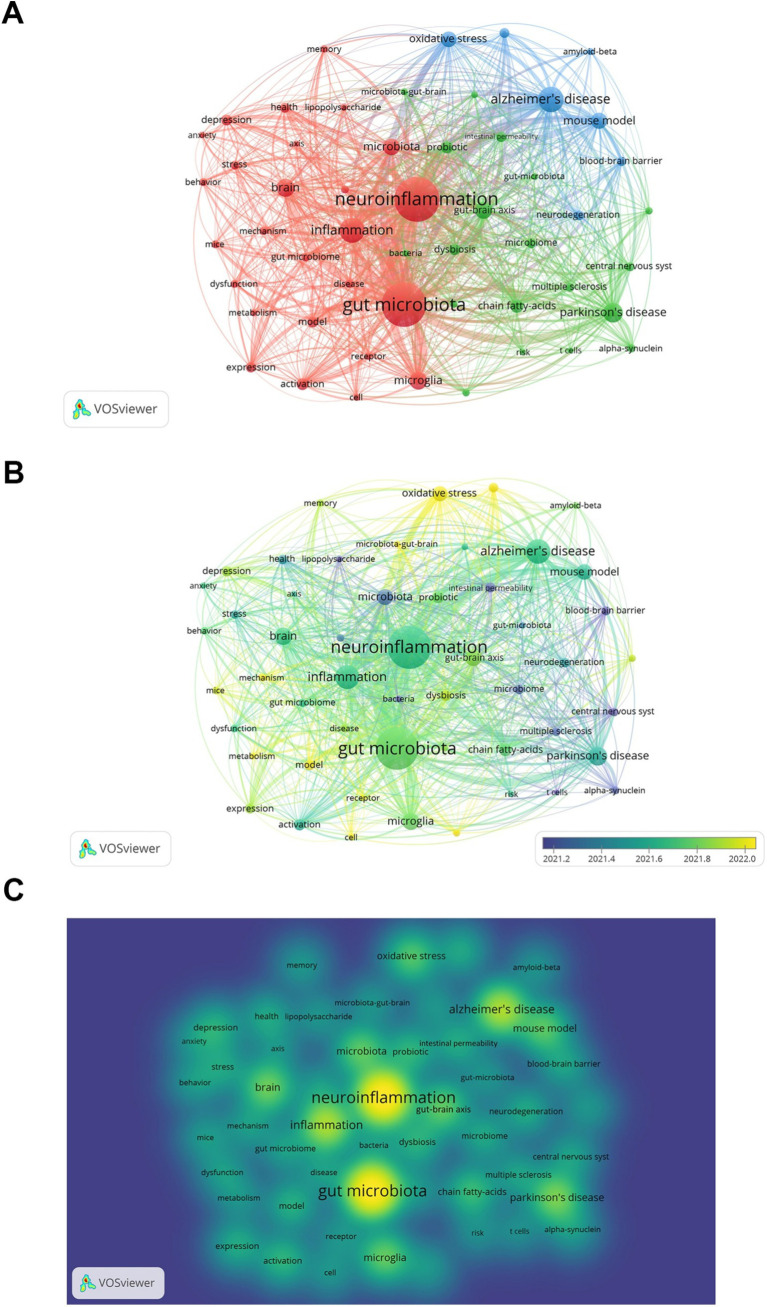
Keyword co-occurrence analysis. **(A)** Keywords network visualization. **(B)** Keywords network visualization with overlaid time. Color represents average time. **(C)** Keywords network density map.

We chose keywords that appeared at least 50 times and carried out a clustering analysis using VOSviewer (Figure 7A). The larger the node was, the more frequently the keyword occurred, and the more it signified a research hotspot. The lines between nodes indicated the strength of association, and the thicker the line was, the stronger the connection between the keywords. As depicted in Figure 7A, we obtained a total of three clusters, representing three research directions. The keywords in the red cluster encompassed neuroinflammation, gut microbiota, and inflammation, among others. The keywords in the green cluster included Parkinson’s disease, gut-brain axis, and chain fatty-acids, among others. The keywords in the blue cluster comprised Alzheimer’s disease, mouse model, and mouse model, among others.

#### 3.6.2 Keywords clustering and timeline graph analysis

Keyword clustering and temporal line analysis are the prominent features of CiteSpace allowing us to have a comprehensive understanding of the hotspots and trends in related research fields. The software parameter settings are as follows: Select the top *N* (*N* = 50) threshold value setting method set the temporal slice to 1 year cluster high-frequency keywords and choose the log-likelihood ratio (LLR) method for clustering marking to generate a clustering view of high-frequency keywords. The statistical results indicate that the Q value is 0.8464 and the S value is 0.9414. With the Q value exceeding 0.3 and the S value surpassing 0.7 it shows that the visualized data is valid and reliable.

**Figure 8:**
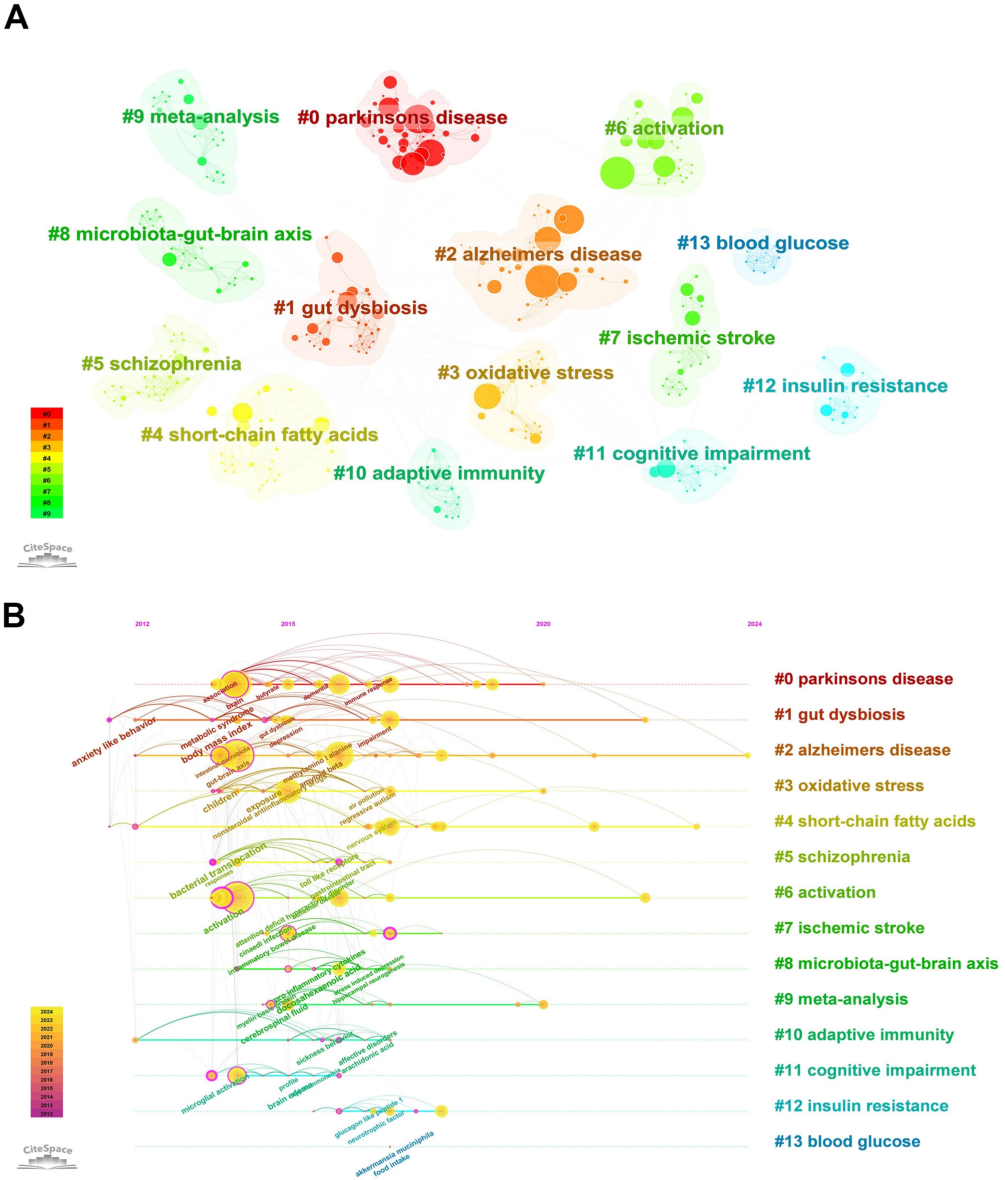
Keywords clustering and timeline graph analysis. **(A)** Cluster analysis of keywords. **(B)** Timeline view of keywords.

Using CiteSpace, a network of keyword co-occurrence was constructed consisting of 445 nodes and 1,608 links (Figure 8A). The software parameters were configured as follows. Time slices: ranging from 2000 to 2024, with each slice representing 1 year. Selection criteria: the top 50 terms with the highest frequency in each slice were selected. Eventually, 13 high-frequency keyword clusters were obtained (Figure 8A; Table 5).

**Table 5:** Cluster analysis of high-occurrence keywords.

Number	Cluster’s name	Top term
#0	Parkinson’s disease	Parkinson’s disease; Alzheimer’s disease; gut inflammation; non-motor symptoms; system
#1	Gut dysbiosis	gut dysbiosis; Parkinson’s disease; psychiatry; quercetin; vitamin d
#2	Alzheimer’s disease	Alzheimer’s disease; gut-brain axis; dementia; Parkinson’s disease; amyloid beta
#3	Oxidative stress	Oxidative stress; autism spectrum disorder; microglia; Parkinson’s; gut microbiota
#4	Short-chain fatty acids	Short-chain fatty acids; mechanisms; fecal microbiota transplantation; microRNA; synaptic plasticity
#5	Schizophrenia	Schizophrenia; immunomodulation; etiology; microbiota interventions; neuroprogression
#6	Activation	Activation; stress; manganese; disease; microglia
#7	Ischemic stroke	Ischemic stroke; mice; lipidomics; metabolomics; social behavior
#8	Microbiota-gut-brain axis	Microbiota-gut-brain axis; refined diet; nanomedicine; traditional Chinese medicine; glutamine metabolism
#9	Meta-analysis	Meta-analysis; neurosteroids; interferon gamma; neopterin; dopaminergic neurons
#10	Adaptive immunity	Adaptive immunity; NMDA receptor; frontal cortex; psychiatric disorders inflammation; psychiatric disorders
#11	Cognitive impairment	Cognitive impairment; bifidobacteria; rat brain; synaptic dysfunction; encephalopathy
#12	Insulin resistance	Insulin resistance; insulin signaling; hippocampus; dysbiosis; fat
#13	Blood glucose	Multiple sclerosis; experimental autoimmune encephalomyelitis; central nervous system; multiple sclerosis; tryptophan

In order to more clearly identify the development context of the research on gut microbiota and neuroinflammation, the keywords were arranged in a chronological sequence and a timeline graph of the keywords was drawn (Figure 8B). Each node represents a distinct research keyword. The size of the node indicates the frequency of the keywords’ occurrence, and the lines represent co-occurrence. The results of keywords clustering from the literature offer us some clues: there are multiple research hotspots in the field of neuroinflammation related to the gut microbiome, such as “Parkinson’s disease,” “Alzheimer’s disease,” “oxidative stress,” “short-chain fatty acids,” and “schizophrenia.”

#### 3.6.3 Keywords burst analysis

**Figure 9:**
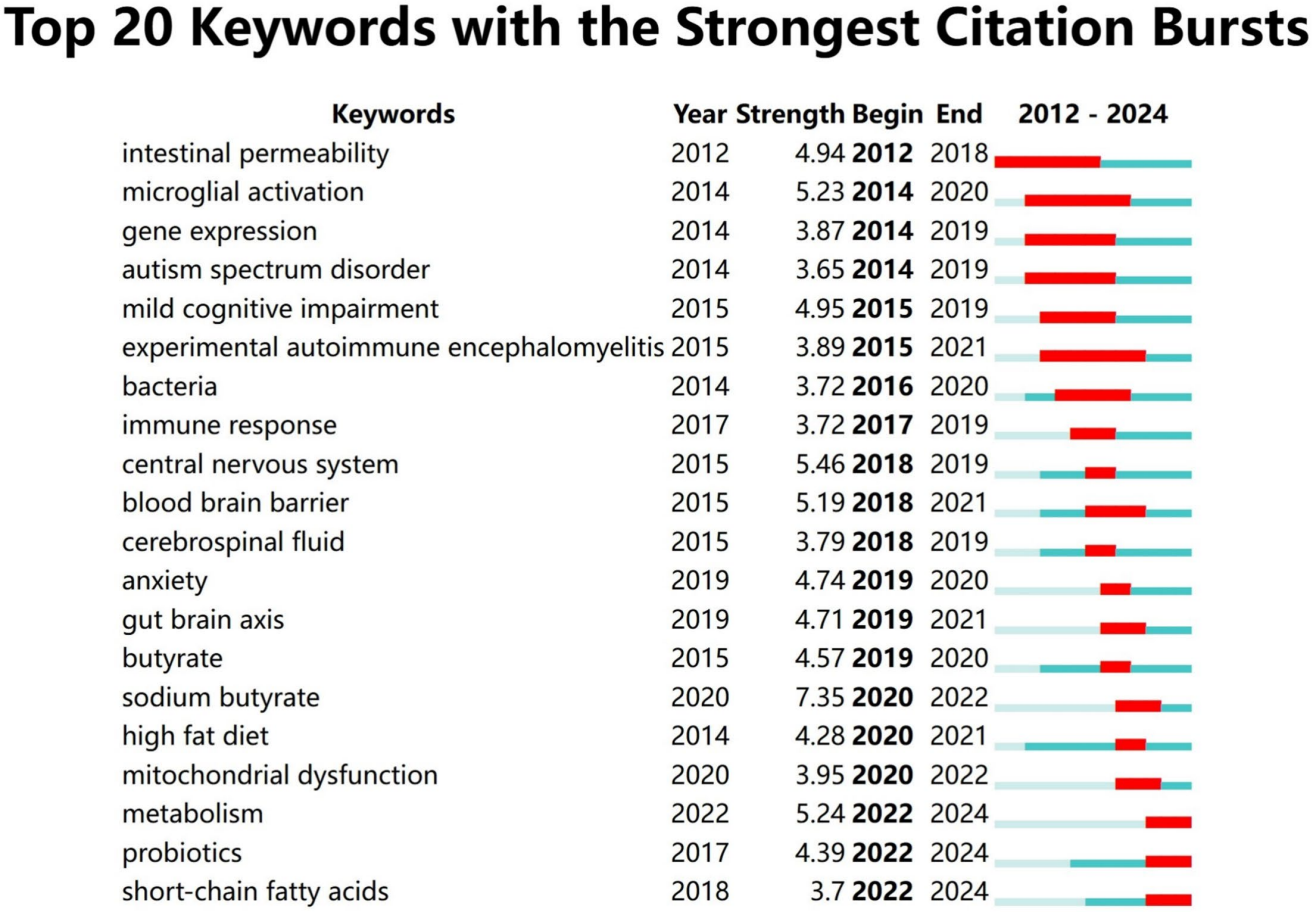
Keywords burst analysis. Top 20 keywords with the strongest citation bursts.

Keywords burst analysis can assist us in comprehending the rapidly escalating research interests or emerging research subjects in a specific field within a particular period and might indicate future research tendencies. We selected the top 20 burst keywords based on burst intensity (Figure 9). The keyword with the highest burst intensity is nitric oxide synthase. The earliest and latest years of keyword bursts are 2012 and 2022, respectively. The keyword with the highest ranking is “sodium butyrate (7.34)” which received extensive attention in 2020 and persisted until 2022. This reflects the researchers’ enthusiasm for exploring the potential role of sodium butyrate in the gut microbiota in neuroinflammation research during that period. The most recent keywords are metabolism (5.29) probiotics (4.43) and short-chain fatty acids (3.73) among which probiotics garnered widespread concern as soon as they emerged which may suggest future research directions.

## 4 Discussion

### 4.1 General information

Since 2000, the number of studies relating gut microbiota to neuroinflammation has presented a consistent upward trend, suggesting that this topic has garnered substantial researcher attention and is in a continuous state of development. Our study offers a comprehensive overview of the increasing interest in this area and identifies the most significant contributors across countries, institutions, journals, and authors. Figure 1 reveals that the number of citations for publications in 2020 peaked, followed by a rapid growth in the number of publications. From the perspective of citation burst analysis, this might be related to the fact that several authoritative journals published systematic reviews in 2020. These representative works integrated the progress in the field and provided theoretical frameworks for subsequent research, significantly increasing the citation volume of the literature that year. For instance, The Lancet Neurology published “The gut microbiome in neurological disorders,” which summarized the potential role of the gut microbiome in multiple neurological disorders over the past decade and pointed out the direction for future research and treatment strategies. The upsurge in research activities since 2020 can be attributed to an enhanced awareness of the role of gut microbiota in CNS-related diseases, which also represents a new milestone of research achievements in this domain.

China and the United States have made remarkable contributions to the research output in this field. China has published a greater number of papers, while the United States has engaged in more extensive inter-institutional collaboration. This might be related to various factors. In terms of policy orientation, China’s “Healthy China 2030 Program Outline” explicitly supports cutting-edge fields such as brain science and microbiome.^1^ The National Institutes of Health (NIH) of the United States has long funded interdisciplinary research on gut microbiota and neuroscience through the “Human Microbiome Project” (HMP) and the “Brain Research through Advancing Innovative Neurotechnologies” (BRAIN Initiative). In terms of the scientific research system, China’s scientific research system is good at concentrating resources to quickly produce results, for example, by accumulating data through large-scale sequencing projects (such as the “Ten Thousand Microbial Genomes Project”), promoting the growth of the number of papers. The American scientific research system, on the other hand, emphasizes cross-institutional collaboration, such as the “National Microbiome Initiative” (NMI) integrating resources from multiple universities, national laboratories, and enterprises. Regarding the authors, Kim, Dong-Hyun (*n* = 12) and Liu, Xuebo (*n* = 11) have published the largest number of papers, indicating their significant contributions to the field. Cryan, JF (*n* = 424), Sampson, TR (*n* = 394), and Erny, D (*n* = 345) are the most highly cited authors, serving as bridges in the field and exerting considerable academic influence. Although many authors have made substantial contributions to this research area, more efforts are necessary to enhance international and domestic institutional cooperation to expedite the solution of major scientific problems and achieve breakthrough progress jointly.

Regarding journals, the top 10 ones accounted for 22.29% of the total number of publications. Over 70% of the publications were presented in 433 periodicals, suggesting that the research topic goes beyond a single focus area and offers a broad perspective and resource selection. This includes top-tier journals like Cell and Nature, demonstrating the innovativeness and academic value of the research topic.

### 4.2 Hotspots and frontiers

Highly frequent co-citation references and keywords often reflect the research focus and direction in a specific field, providing valuable insights into important findings. We used key co-occurrence analysis to identify the main research focus and emerging trends in the study of the association between gut microbiota and neuroinflammation, and to clarify the evolution and dynamics of its subject structure.

#### 4.2.1 Relationship between gut microbiota metabolism and neuroinflammation

According to Table 3, a study published by Sampson et al. (2016) in Cell in 2016 possesses the highest citation burst intensity, signifying its significant breakthrough and receiving substantial attention from relevant researchers. The study indicated that the gut microbiome regulates neuroinflammation and *α*-synuclein aggregation via a pathway that affects the nervous system, modulating neuroinflammation in PD models and uncovering the crucial relationship between the gut microbiome and the neuroinflammation pathology of Parkinson’s disease. The review article titled “THE MICROBIOTA-GUT-BRAIN AXIS” written by John F. Cryan et al. comprehensively reviews the research progress of MGBA. It discusses how the gut microbiota affects brain functions and behaviors through multiple pathways, thereby influencing the host’s physiological, behavioral and cognitive functions, and playing a significant role in various neuropsychiatric disorders (Cryan et al., 2019). This study not only provides a theoretical basis for understanding how the gut microbiota affects neuropsychiatric health, but also offers a new perspective for future research directions. The paper titled “Host microbiota constantly control maturation and function of microglia in the CNS” published by Daniel Erny in Nature Neuroscience investigated the influence of the host microbiota on microglia in the CNS. It was discovered that the microbiota regulates the maturation and function of microglia through metabolites like SCFAs. The absence of the microbiota would result in abnormal microglia, while reintroducing a complex microbiota could partially restore their normal characteristics.

MGBA plays a vital role in facilitating bidirectional communication between the gut microbiome and the brain, being capable of maintaining and promoting beneficial microorganisms, which are indispensable for maintaining normal functions (Grosso, 2021; Margolis et al., 2021). MGBA involves complex interactions between multiple systems, including the immune system related to the gut (Minaya et al., 2024), the enteric nervous system (Luesma et al., 2024), the neuroendocrine system (Yu et al., 2024), and the gut microbiome itself. These interactions are promoted by the gut microbiome, which generates crucial compounds such as neurotransmitters (Wu et al., 2024), tryptophan (Gu et al., 2024), and SCFAs (Shen et al., 2024), etc. These compounds simultaneously exert influences on the functions of both the gut and the brain. Furthermore, the metabolites of the gut microbiome, for instance, SCFAs, have been verified to be capable of traversing the BBB and thereby influencing the neurochemical milieu of the brain (Mann et al., 2024; Uceda et al., 2023). The gut microbiome assumes a key role in preserving the integrity of the intestinal barrier, facilitating the prevention of the infiltration of pathogens and toxins and thereby forestalling the occurrence of systemic inflammation. Nevertheless, compromised intestinal barrier function can result in augmented intestinal permeability, namely the so-called “leaky gut, “enabling harmful substances to enter the bloodstream and subsequently impact brain health (Aburto and Cryan, 2024).

These findings further substantiate the notion that the gut microbiome plays a crucial role in regulating neuroinflammation. Hence, a more profound comprehension of the interactions between the gut microbiome and neuroinflammation is of paramount importance for developing novel therapeutic strategies and preventive measures.

#### 4.2.2 Neuroinflammation and other disorders related to the gut microbiota

Modern research has disclosed that alterations in the composition of the gut microbiome might influence neuroinflammation through diverse mechanisms, thereby facilitating the advancement of diseases such as AD, PD, and MS. From the results of cluster analysis, it can be seen that there are some common mechanisms underlying the association between gut microbiota and the pathological manifestations of neuroinflammation in these diseases. For instance, there are disorders of intestinal barrier function, the cross-action of oxidative stress (OS), and the bidirectional regulation of short-chain fatty acids (SCFAs). Some researchers have compared the fecal microbiome of AD patients to that of healthy controls and discovered substantial differences between the two. The dysregulation of MGBA could result in neuropathological alterations and cognitive dysfunction in AD patients, encompassing neuroinflammation, increased BBB permeability, and exacerbated Aβ deposition (Jin et al., 2023; Krishaa et al., 2023). It was further confirmed the connection between the abundance of gut bacteria and the markers identified in the cerebrospinal fluid of AD patients (Xie et al., 2023). The transplantation of the fecal microbiota from normal mice into AD model mice can ameliorate the cognitive function, Aβ pathology and hippocampal inflammation of these mice (Shen et al., 2020), further suggesting that the disorder of gut microbiota in AD patients is associated with the increased generation of cerebral amyloid beta proteins.

Studies have demonstrated that neuroinflammation and Oxidative Stress (OS) play a significant role in the neuronal damage of PD patients. The content of metabolites produced by gut microbiota can vary, which can influence neuroinflammation and OS through the MGBA and be involved in the progression of PD. For instance, one of the common endotoxins in gut microbiota, LPS, may enhance its penetration in the intestinal wall. This may be associated with neuroinflammation and OS in PD patients and may further exacerbate neuronal damage (Massaro Cenere et al., 2024). Additionally, certain gut bacteria can produce SCFAs, which play a crucial role in regulating immune responses and maintaining the integrity of the gut barrier (Silva et al., 2020). Changes in SCFAs levels may be associated with the neuroinflammatory state in PD patients, as they can affect the differentiation and function of T cells, thereby influencing the regulation of neuroinflammation (Gao et al., 2024).

MS is a chronic inflammatory disease that demyelinates the CNS (Kalia et al., 2024). In common animal models of MS, alterations in the microbiome have also been witnessed (Jangi et al., 2016). The intestinal microenvironment possesses the capacity to regulate the activation and differentiation of self-reactive T cells and direct them towards the CNS (Miljković et al., 2021). When the permeability of the intestine varies, the quantity of harmful or immunogenic antigens passing through the mucosa rises, which might result in persistent neuroimmune dysregulation (Camara-Lemarroy et al., 2018). Numerous clinical and experimental research findings indicate that intestinal microbial dysregulation might be involved in the occurrence of MS through multiple means, such as regulating the differentiation of T lymphocytes, enhancing the permeability of the BBB, and modulating immune factors (Fettig et al., 2024). At present, further fundamental research is still required to clarify the mechanisms underlying these correlations.

As the research advances, the influence of the gut microbiome and neuroinflammation on related diseases becomes more evident, offering a potential for the development of novel treatment strategies. Future research is required to further clarify the specific interaction mechanisms between the gut microbiome and neuroinflammation, as well as how to improve the clinical symptoms and prognosis of neurodegenerative diseases through intervention in the gut microbiome.

#### 4.2.3 Treatments related to neuroinflammation and gut microbiota

The imbalance of specific microbiota may intensify the inflammatory response in the CNS, and subsequently facilitate the development of neurodegenerative changes (Wei et al., 2024). Probiotics are live microorganisms that are beneficial to the human body and have been proven to play a crucial role in treating autoimmune diseases and regulating dysbiosis in the gut. For instance, supplementing with probiotics and dietary fiber has demonstrated potential anti-inflammatory and neuroprotective effects. Research has discovered that interventions utilizing specific strains of lactic acid bacteria and bifidobacteria have shown great prospects in correcting ecological imbalances, reducing neuroinflammation, and enhancing motor function in PD models (Domínguez Rojo et al., 2024).

Probiotic therapy also has a positive effect on regulating the relationship between stress, neuroinflammation, and irritable bowel syndrome (Bruce-Keller et al., 2018). These drugs include hormones such as serotonin, gastrin, and progesterone, ANS regulators such as beta-receptor blockers, lipid-lowering drugs such as statins, and gut microbiome regulators such as probiotics and antibiotics, which target different inflammation pathways in the brain and gut after trauma to reduce neuroinflammation (Azarfarin et al., 2024). These therapeutic drugs have a promising future in reducing inflammatory responses in the MBGA and improving neurocognitive abilities in patients with traumatic brain injury. The gut microbiome also plays a regulatory role in the motor deficits and neuroinflammation in PD models, with SCFAs playing a key role. The research results suggest that changes in the gut microbiome may represent a new risk factor for PD, and future possible treatment options may include targeting immune activation through the microbiome (Wang et al., 2024).

Fecal microbiota transplant (FMT) can improve the diversity and function of the gut microbiome. This therapeutic approach has shown positive effects in treating certain intestinal diseases, such as refractory Crohn’s disease (Kao et al., 2024) and recurrent Clostridioides Difficile Infections (CDI) (Ghani et al., 2024).

In the context of neuroinflammation, FMT may indirectly have a positive impact on neuroinflammation by affecting the host’s immune system and metabolic pathways. Research has found that SCFAs have significant differences before and after FMT treatment for CDI, which can significantly improve cognitive function, emphasizing the need for clinicians to be aware of the impact of FMT on MGBA and its potential as a treatment for dementia patients (Park et al., 2021). Additionally, Zhao et al. (2021) have discovered that FMT is capable of alleviating the damage of the BBB and suppressing neuroinflammation in the substantia nigra, thereby diminishing the injury of dopaminergic neurons. A double-blind, placebo-controlled, randomized clinical trial result shows that fecal microbiota transplantation (FMT) is safe for patients with Parkinson’s disease, but it does not provide clinically significant improvements (Scheperjans et al., 2024). Probiotics and FMT have shown potential in neuroinflammatory-related diseases, but current evidence mostly comes from small-scale trials and animal studies. Their application is still at the initial stage of research, and it is necessary to solve issues such as strain specificity, individualized responses, and long-term safety before they can be translated to clinical practice.

In recent years, the advantages of traditional Chinese medicine in regulating intestinal microbiota to improve neuroinflammation have gradually become apparent. In the field of research on single herbs, it has been found that low-fructose polygalactoside from Morinda officinalis can increase the abundance, diversity, and stability of beneficial bacteria, thereby influencing the shape of the intestine, the production of mucus, and the permeability of the intestinal mucosa. Additionally, it can improve OS state and downregulate the expression of Aβ1-42, thereby helping to slow down the pathological progression of AD (Chen et al., 2017). Rhein is capable of treating cognitive dysfunction in high-fat diet-induced mouse models of hyperlipidemia by regulating the homeostasis of the microbiome, inhibiting the accumulation of macrophages, counteracting neuroinflammation, and promoting the expression of BDNF (Wang et al., 2016). There are also studies that show that quercetin can significantly improve cognitive function when vitamin D levels are low, reduce the accumulation of Aβ, tau protein phosphorylation, and neuroinflammation, and this improvement may be related to enhancing the diversity and richness of the intestinal microbiome (Lv et al., 2018). In terms of compound research, traditional Chinese medicine, centering on the latest pathogenic mechanism theory of intestinal microbiota and neuroinflammation, has put forward traditional Chinese medicine compounds with multi-pathway and multi-target therapeutic effects, such as Jiedu Huayu Decoction (Zhang et al., 2019), Chaihu Shugan Powder (Hu et al., 2020), Danggui Shaoyao Powder (Pan et al., 2022) and Xiaoyao Powder (Hao et al., 2024). By regulating the microecology of the digestive tract, they directly or indirectly suppress inflammatory responses and activate the immune response in the brain, thereby improving neuroinflammation. Despite the potential of TCM in treating neuroinflammation, its mechanism of action is complex, involving multiple signaling pathways and multiple targets. Therefore, future research needs to further investigate the specific effects of TCM on the gut microbiome, as well as how these effects translate into therapeutic effects against neuroinflammation.

In conclusion, the relevant therapeutic modalities of the gut microbiome and neuroinflammation not only facilitate a deeper comprehension of the mechanisms of neuroinflammation but also offer novel strategies and targets for the prevention and treatment of neurodegenerative disorders. Future studies need to explore in depth the specific mechanisms between the microbiome and neuroinflammation and develop targeted microbiome modulation therapeutics, personalized medicine and precise nutrition will emerge as significant directions in the treatment of neurodegenerative diseases. Nevertheless, this research has certain limitations. For instance, the selection of a single WoSCC database and the time constraint might result in some information bias. Future research needs to break through the limitations of a single database and integrate PubMed, Scopus, clinical cohort data, and multi-omics (metagenomics, metabolomics, proteomics) information to comprehensively analyze the molecular network of microbiota-host interaction. At the same time, innovative research methods should be introduced, such as the incorporation of machine learning, complex network modeling, and causal inference methods, to reveal the causal relationship between the phased characteristics of neuroinflammation and the evolution of microbiota.

## 5 Conclusion

This study offers a visual overview of the research on the association between gut microbiota and neuroinflammation through bibliometric analysis, delving into their relationship, the mechanisms influencing the pathogenesis of related diseases, and the potential value of corresponding therapeutic approaches. This provides a rather comprehensive perspective for understanding the connection between gut microbiota and neuroinflammation and supplies ideas and references for future in-depth studies. The future should break through the limitations of a single database and introduce more innovative research methods to gain a deeper understanding of the interaction between gut microbiota and neuroinflammation, as well as their potential applications in disease treatment, thereby providing more precise guidance for clinical treatment.

## Data Availability

The original contributions presented in the study are included in the article/supplementary material, further inquiries can be directed to the corresponding author.

## Footnotes

^1^ https://www.gov.cn/zhengce/2016-10/25/content_5124174.htm
